# Flow diverter treatment for proximal middle cerebral artery non-saccular aneurysms: multicenter efficacy and safety analysis

**DOI:** 10.3389/fneur.2025.1586956

**Published:** 2025-07-14

**Authors:** Hao Yao, Chao Zou, Shijie Zhu, Yina Wu, Jianfei Sun, Zhiwen Lu, Qinghai Huang

**Affiliations:** ^1^Department of Neurosurgery, Jinjiang Municipal Hospital, Quanzhou, Fujian, China; ^2^Neurovascular Center, Changhai Hospital, The Naval Medical University, Shanghai, China; ^3^Department of Neurosurgery, Naval Medical Center, The Naval Medical University, Shanghai, China

**Keywords:** intracranial aneurysm, middle cerebral artery, endovascular treatment, flow diverter, digital subtraction angiography

## Abstract

**Objective:**

Proximal middle cerebral artery (MCA) non-saccular aneurysms present unique therapeutic challenges due to their morphology and proximity to critical branches. This study evaluates the safety, efficacy, and technical nuances of flow diverter (FD) devices in treating these lesions, with a focus on optimizing device selection and perioperative management.

**Methods:**

A retrospective multicenter analysis included 43 patients with M1/M2 segment non-saccular aneurysms treated with FD between 2020–2024. Perioperative antiplatelet regimens were individualized based on platelet function assays and genotype analysis for CYP2C19 polymorphisms. Procedural outcomes, complications, and angiographic results were assessed. Virtual stent simulation was utilized in 62.8% of cases for preoperative planning.

**Results:**

Flow diverter (FD) implantation achieved 100% technical success. Perioperative complications occurred in 4.7% (2 transient deficits, 1 hemorrhage). Follow-up angiography (median 8.4 months; *n* = 38) demonstrated 92.1% complete occlusion (OKM-D), with 7.9% partial occlusion. In-stent stenosis occurred in 3 cases (7.9%), all asymptomatic. Clinical follow-up (median 25 months) revealed 97.7% favorable outcomes (mRS ≤ 2). Covered branches (M2, anterior temporal artery) exhibited stenosis in 23 cases and occlusion in 5, none clinically significant.

**Conclusion:**

Flow diverter (FD) therapy for proximal MCA non-saccular aneurysms achieves high occlusion rates with low morbidity, particularly when combined with preoperative simulation and genotype-guided antiplatelet regimens. This study suggests that FD devices may serve as a potential alternative for traditional surgical treatment for this kind of aneurysms.

## Highlights

While FD therapy is well-established for internal carotid artery aneurysms, its use in the MCA—a region with complex anatomy and functional significance–remains understudied. Our work provides robust clinical data on FD efficacy and safety in this high-risk territory, demonstrating a 100% technical success rate, an 4.7% perioperative complication rate, and an 92.1% complete occlusion rate at mid-term follow-up.

## Introduction

The middle cerebral artery (MCA) is a common site for intracranial aneurysms due to its complex anatomy, proximity to functional brain regions, and role in supplying blood to the cerebral hemispheres (1, 2). While surgical clipping remains a cornerstone for MCA aneurysm management, non-saccular aneurysms (e.g., fusiform or dissecting types) pose significant challenges due to their intricate morphology and rapid progression. Flow diverters (FDs), designed to reconstruct the parent artery and induce aneurysm thrombosis through hemodynamic modulation, have revolutionized the treatment of complex intracranial aneurysms (3). However, FD application in the MCA is complicated by its anatomical features (e.g., variable bifurcation patterns, small vessel caliber) and concerns regarding branch occlusion (4). This study evaluates the safety and efficacy of FD therapy for proximal MCA non-saccular aneurysms and explores factors influencing clinical outcomes.

## Materials and methods

### Study population

This study is a retrospective analysis involving 43 patients with proximal MCA non-saccular aneurysms treated with flow diversion (FD) at three centers from January 2020 to June 2024. The inclusion criteria were: (1) diagnosis of MCA non-saccular aneurysms (M1-M2 segment) via digital subtraction angiography (DSA); (2) receipt of FD treatment, regardless of whether coiling was used; (3) availability of complete clinical and imaging data. The exclusion criteria were: (1) aneurysms in the M3 segment or distal MCA; (2) saccular aneurysms at the bifurcation of the MCA; (3) recurrent aneurysms that had previously undergone stent-assisted embolization. The MCA segmentation was based on the classification by Gibo et al. (5). Proximal MCA non-saccular aneurysms were diagnosed when DSA images illustrated fusiform or irregular dilations in the M1 or M2 segments of the MCA. These lesion segments typically exhibited the double lumen sign, rosette sign, or string sign, with no branches arising from the aneurysmal sac or neck (6). The study was approved by the hospital ethics committee, and all patients provided informed consent.

### Endovascular procedure

All procedures were performed under general anesthesia via femoral access. Systemic heparinization was maintained throughout the procedure. FD sizing was based on aneurysm morphology, neck dimensions, and parent artery diameter. An optimal working angle was selected to ensure clear visualization of the proximal and distal vascular pathways, facilitating intraoperative evaluation of stent deployment and apposition. For larger aneurysms or cases with significant discrepancies in proximal and distal vessel diameters, the workstation software or other commercial tools were employed to simulate stent deployment, aiding in FD selection. The FD brand was selected based on aneurysm type, neck size, parent vessel diameter, and device availability during the treatment period. Guided by patient-specific vascular anatomy, long sheaths, intermediate catheters, or guiding catheters were chosen to ensure adequate support for the FD delivery microcatheter. Standard microguidewires and FD-compatible microcatheters were employed to deliver the FD to the aneurysm's distal end. A following fluoroscopic confirmation of positioning, the FD was deployed, and flat-panel computed tomography angiography (FPCTA) was performed intraoperatively or immediately post-deployment to verify stent apposition. Adjunctive coiling was performed concurrently when indicated by aneurysmal morphology.

### Virtual visualization of FD device deployment

AneuGuide™ software optimizes FD deployment for proximal MCA non-saccular aneurysms, particularly in cases involving large aneurysms or significant vessel diameter discrepancies. The workflow initiates with 3D vascular reconstruction, where pre-operative angiographic images are processed to generate a patient-specific 3D vascular model. Subsequently, landing zone definition is performed: the region of interest is segmented, a vascular centerline is derived using maximal inscribed spheres, and proximal/distal landing zones are selected along this centerline. Finally, virtual FD simulation visualizes deployments of different FD models and sizes while mapping critical hemodynamic parameters—including wall apposition, metal coverage, and pore density—to guide optimal device selection. This fully automated process is completed within several minutes, enabling efficient intraoperative navigation and procedural optimization. Further details can be found in the previous study by Tong et al. (7).

### Perioperative antiplatelet management

Preoperative dual antiplatelet therapy with aspirin (100 mg/day) and clopidogrel (75 mg/day) was administered for 5 to 7 days in the vast majority of unruptured cases. Conversely, only one patient with a ruptured aneurysm received loading doses of aspirin and clopidogrel, each at 300 mg, 2 h before the procedure. All patients underwent routine CYP2C19 genotyping via PCR-RFLP (Xiamen Zeesan Biotech Co.) and received thromboelastography to assess platelet function. Patients with CYP2C19 loss-of-function alleles (^*^2/3) or adenosine diphosphate inhibition <30% received ticagrelor (90 mg twice daily) (8). Postoperatively, dual antiplatelet therapy (aspirin and clopidogrel or ticagrelor) was maintained for 3 months, followed by continuous aspirin monotherapy.

### Clinical and angiographic outcome assessment

Patients underwent clinical follow-up assessments pre-discharge, at 3 months, 6 months, and annually thereafter. New neurological deficits were systematically documented at each interval. Postoperative clinical outcomes were evaluated using the modified Rankin Scale (mRS), with a score of ≤ 2 defined as a favorable prognosis. Digital subtraction angiography (DSA) was performed at 6 months post-procedure to assess aneurysm occlusion via the O'Kelly-Marotta (OKM) grading system: Grade A = complete aneurysm filling (>95%); Grade B = partial filling (5–95%); Grade C = neck remnant (<5%); Grade D = complete occlusion (9). Covered branch patency was classified into 4 categories: (1) Unobstructed: Smooth blood flow; (2) Narrowed without flow impairment: Stenosis at branch origin with preserved flow velocity; (3) Narrowed with flow impairment: Stenosis at branch origin with reduced flow velocity; or (4) Occluded: Absent perfusion. In-stent neointimal hyperplasia was graded as: 0 = none; 1 = mild (<25%); 2 = moderate (25%−50%); 3 = severe (>50%). Patients with incomplete aneurysm occlusion (OKM grades A–C) at 6 months underwent additional follow-up DSA at 12 months.

## Results

### Baseline characteristics

The cohort comprised 28 males and 15 females (mean age: 52.1 ± 6.5 years). Clinical presentations included headache in 21 cases (1 case being a preoperatively ruptured aneurysm), focal neurological deficits in 3 cases, and seizure onset in 1 case, with 18 patients having aneurysms discovered incidentally. A history of hypertension was present in 25 cases, and a history of cerebral infarction in 12 cases. Aneurysms were located in the M1 segment (36 cases), M2 segment (4 cases), and overlapping M1–M2 (3 cases), with a mean diameter of 7.9 ± 2.1 mm, ranging from 6–25 mm. Nine patients had additional non-MCA saccular aneurysms. Preoperative virtual stent simulations were utilized for 27 cases (62.8%) with complex anatomy.

### Procedural outcomes

**Figure 1:**
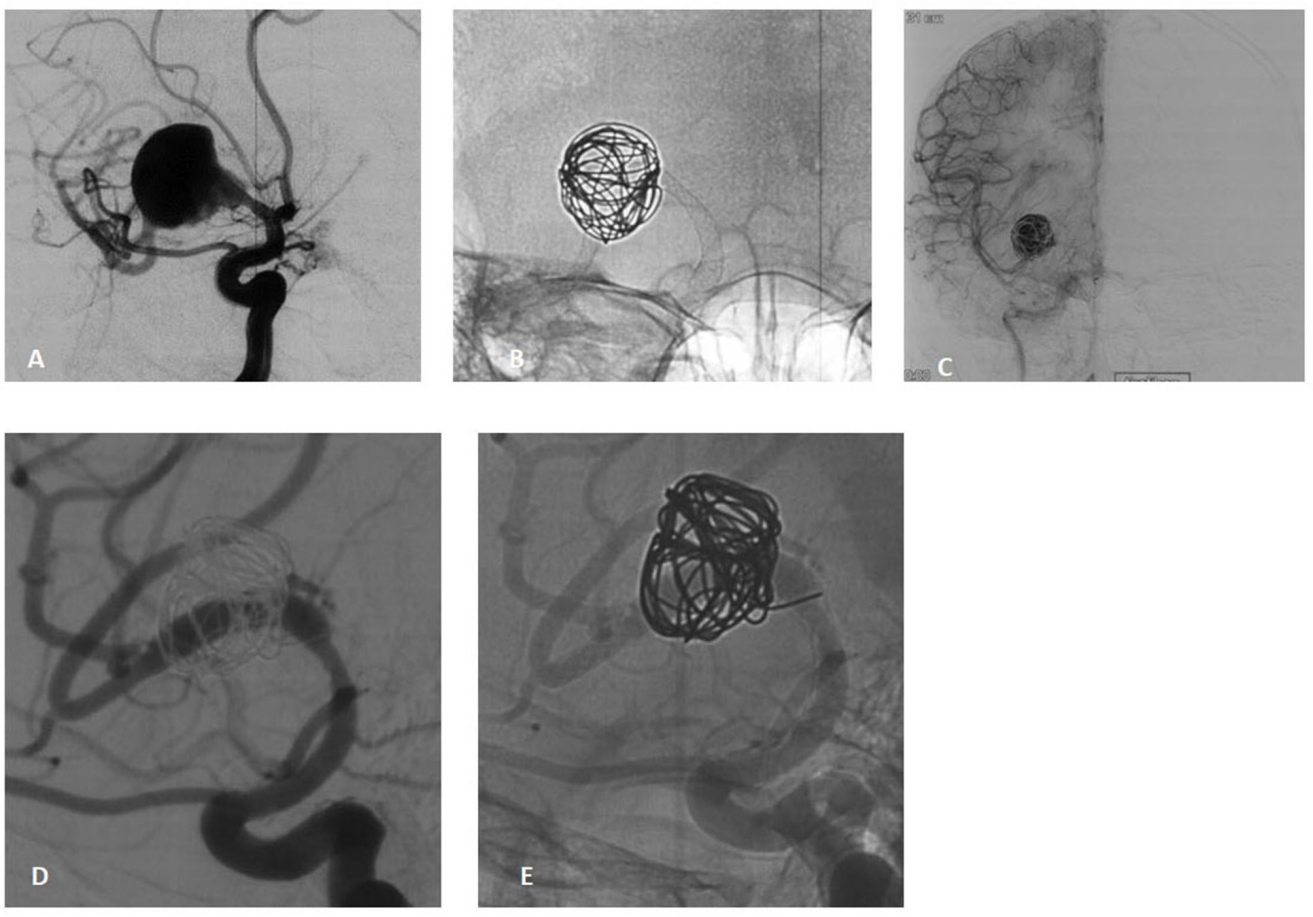
A 38-year-old male presenting with acute-onset headache and blurred vision due to a large dissecting aneurysm in the right M1 segment. **(A)** Pre-procedural angiography reveals a large dissecting aneurysm in the M1 segment. **(B)** Pipeline Flex 3.75/30 deployment with loose coil packing within the aneurysm sac. **(C)** Immediate post-procedural angiography demonstrates significant contrast stagnation in the aneurysm and patency of the parent artery. **(D, E)** Eight-month follow-up DSA confirms complete aneurysm healing (OKM grade D), patent parent artery, and mild stenosis at the origin of the superior M2 branch without flow impairment.

**Figure 2:**
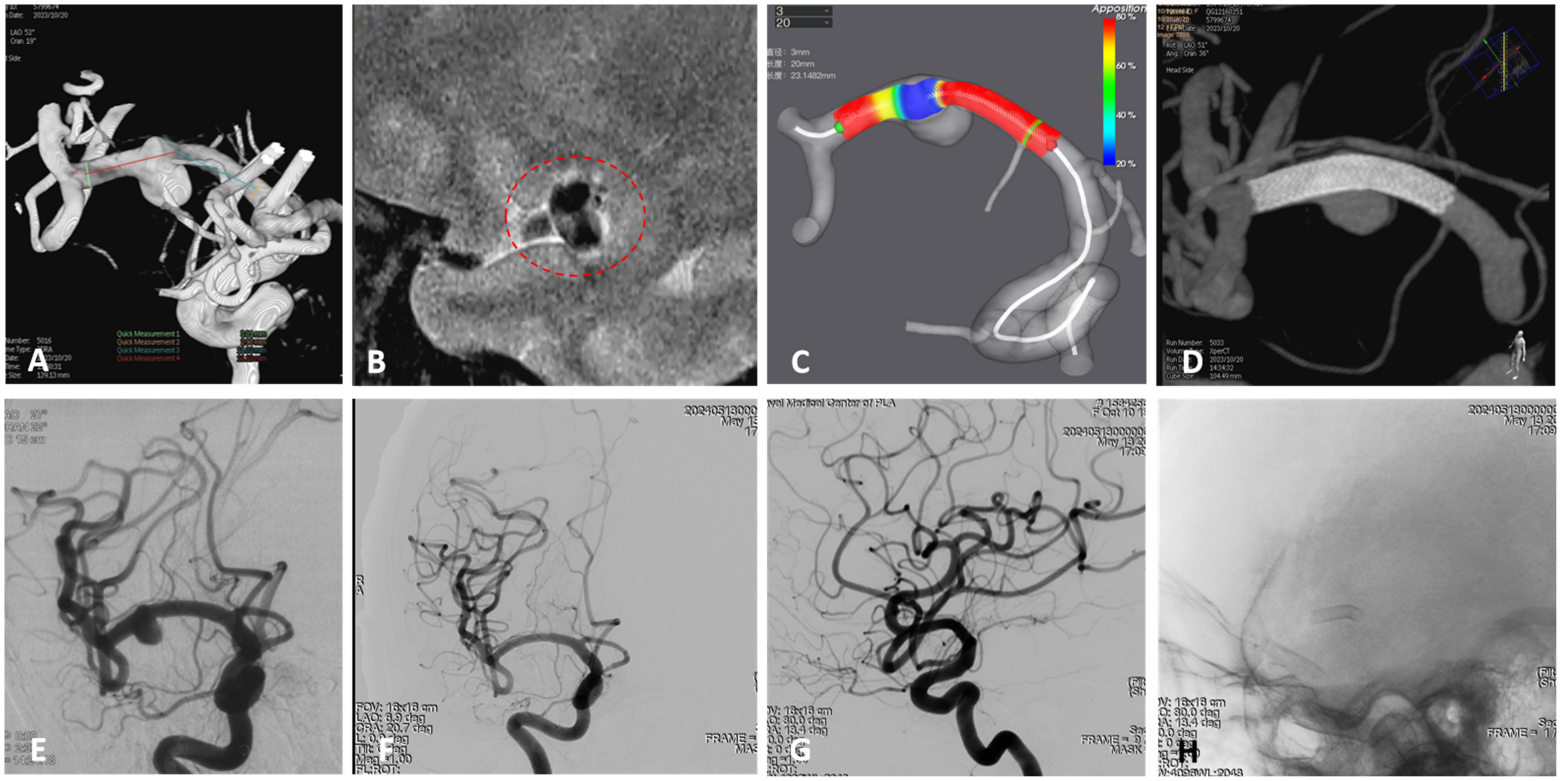
A 59-year-old female presenting with headache was diagnosed with a right middle cerebral artery (MCA) non-saccular aneurysm. **(A)** Digital subtraction angiography (DSA) revealed dilation of the M1 segment of the right MCA (arrow). **(B)** High-resolution magnetic resonance imaging (HR-MRI) showed partial enhancement of the aneurysm wall (circle). **(C)** Pre-procedural simulation of flow diverter (FD) deployment using AneuGuideTM software (Hangzhou Arteryflow Technology Co., Ltd.) to optimize device sizing and positioning. **(D)** Immediate postoperative Vaso computed tomography (CT) indicated good wall apposition of the FD (Lattice 2.9/20), without coverage of perforating branches. Preoperative DSA confirming aneurysm morphology and parent vessel anatomy. **(E)** Immediate postoperative DSA confirmed partial contrast filling of the aneurysm sac, with patency of the parent vessel. **(F–H)** Follow-up angiography at 7 months demonstrated complete remodeling of the right MCA, complete occlusion of the aneurysm, and patency of the lenticulostriate artery.

FD implantation was successful in all cases, with one patient having both M1 and M2 segments affected, necessitating the use of 2 FD devices for bridging due to a longer lesion. Another preoperatively ruptured MCA dissection, with 2 overlapping FD devices deployed in the parent artery and coiling performed within the aneurysm sac. Adjunctive coiling was performed in 7 large/giant aneurysms (Figure 1). The remaining patients were treated with a single FD, as illustrated in Figure 2. A total of 45 FDs were implanted in 43 aneurysms, including 20 Tubridge, 18 Pipeline Flex, 5 Lattice, 1 Surpass Evolve, and 1 Nuwa FD. Two patients experienced challenges with microcatheter passage due to severe stenosis of the parent vessel and underwent the procedure of balloon angioplasty for pre-dilation. Immediate postoperative angiography showed that all patients maintained patency in the feeding arteries and visible branches. Based on the OKM grading system, 32 cases were classified as A, 10 cases as B, and 1 case as C.

### Complications

One patient with an M2 segment dissecting aneurysm underwent Tubridge FD implantation and recovered uneventfully initially. However, 2 h postoperatively, the patient developed transient dysarthria and left-sided hemiparesis. Immediate computed tomography (CT) angiography revealed no hemorrhage or structural abnormalities, and symptoms resolved spontaneously within 10 min. The patient was discharged neurologically intact following continued dual antiplatelet therapy (ticagrelor and aspirin). A second patient experienced sudden-onset headache and left-sided hemiparesis 3 weeks post-procedure. CT imaging demonstrated cortical hemorrhage progressing to subdural hematoma and cerebral herniation, necessitating emergent craniotomy for hematoma evacuation and decompressive craniectomy. At the 6-month follow-up, this patient exhibited moderate disability (modified Rankin Scale [mRS] score of 3). A third patient developed a basal ganglia infarction secondary to lenticulostriate artery occlusion. Two additional non-neurological complications occurred, both involving groin puncture site hematomas. All cases received prompt intervention without permanent neurological sequelae.

### Follow-up

All patients were clinically followed up for a median of 25 months (range: 6 months to 4 years). No aneurysm ruptures or deaths occurred during this period. Excluding the previously mentioned hemorrhagic cases, the remaining patients recovered well. A total of 38 patients underwent follow-up DSA at an average of 8.4 months, revealing a complete occlusion rate of 92.1% (grade D) and a partial occlusion rate of 7.9% (grade C). Three cases exhibited significant in-stent stenosis. The covered branches included M2 branches of the MCA, anterior temporal artery branches, choroidal anterior artery, and anterior cerebral artery. Among these, 23 cases had stenosis at the covered branch openings, and 5 cases had occlusion, all without associated clinical symptoms.

## Discussion

Proximal MCA non-saccular aneurysms (e.g., fusiform or dissecting subtypes) pose significant therapeutic challenges due to the absence of a defined neck, involvement of elongated vascular segments, and high risks of ischemic stroke with microsurgical techniques or recurrence with stent-assisted coiling (10, 11). Traditional endovascular methods are further complicated by branch vessel occlusion risks and thrombotic potential from overlapping stents, leaving optimal treatment strategies uncertain. Our multicenter study demonstrates that flow diversion represents a highly effective therapeutic strategy for proximal MCA non-saccular aneurysms, achieving 100% technical success and 92.1% complete occlusion (OKM-D) at mid-term angiographic follow-up. Despite frequent branch coverage (stenosis: 53.5%; occlusion: 11.6%), no clinical sequelae have occurred. The 97.7% favorable clinical outcomes (mRS ≤ 2) at 25-month follow-up further validate the safety-efficacy balance of this approach. Notably, the low perioperative complication rate (4.7%) and asymptomatic nature of in-stent stenosis (7.9%) contrast favorably with historical risks of microsurgery or stent-assisted coiling. These results suggest that individualized antiplatelet regimens and precise FD sizing may mitigate thrombotic risks while optimizing hemodynamic reconstruction.

The use of FDs for intracranial aneurysm treatment has gained recognition over recent years due to accumulating evidence supporting their safety and efficacy (12). However, most evidence originates from studies of internal carotid artery (ICA) aneurysms and those located within the Circle of Willis. Despite technological advancements in FD design for distal cerebral vessels, the MCA poses unique challenges due to its anatomical complexity and critical role in supplying motor and language cortical regions. FD implantation risks insufficient perfusion of these functional areas, potentially leading to transient or permanent neurological deficits. Additionally, significant variability in MCA branching patterns—such as bifurcations and trifurcations—complicates treatment (13). FD placement in M1/M2 segments frequently results in branch coverage, with outcomes influenced by collateral circulation and the rate of branch occlusion. Preclinical studies by Kallmes et al. demonstrated long-term patency in 88% of covered branches alongside complete aneurysm occlusion, attributed to persistent branch flow vs. stagnation at the aneurysm dome (14). While lenticulostriate artery coverage during MCA aneurysm treatment has not been associated with increased clinical symptoms, likely due to limited basal ganglia collateralization, maintaining physiological perfusion to MCA-dependent territories remains challenging. Collateral leptimeningeal vessels may supply distal regions of covered branches, but competitive collateral flow reduces antegrade pressure gradients, potentially accelerating branch occlusion (15). Currently, no reliable methods exist to predict post-FD branch vessel fate. Therefore, precise assessment of FD landing zones in unaffected vessel segments and minimization of normal branch coverage are critical to mitigate postoperative ischemic risks.

Beyond concerns related to branch vessel coverage, the efficacy of FD treatment for aneurysms of MCA remains debated. Notably, anatomical distinctions between bifurcation saccular aneurysms and non-saccular aneurysms at non-bifurcation sites may yield divergent outcomes. Branch occlusion during flow diversion may influence aneurysm prognosis (16). Topcuoglu et al. investigated FD-induced branch coverage and aneurysm occlusion rates, demonstrating that saccular aneurysms had a 40% occlusion rate compared to 75% for fusiform aneurysms (17). The authors proposed FD as the preferred endovascular therapy for fusiform, dissecting, or cortical branch MCA lesions but cautioned against its use for bifurcation saccular aneurysms. To date, experimental bifurcation aneurysm studies lack mid-term follow-up data on FD efficacy under single or combined device strategies (18). Diestro et al. reported favorable occlusion rates in 54 FD-treated MCA bifurcation aneurysms but noted a 17% thromboembolic complication rate (2). Similarly, Caroff et al. observed incomplete occlusion in 38% of FD-treated MCA bifurcation aneurysms and a 21% treatment-related adverse event rate, concluding that FD is not recommended for this subtype (19). A systematic review of FD use in small-vessel aneurysms further reported lower complete occlusion rates for saccular aneurysms (55%) compared to non-saccular aneurysms (73%) (20). These findings suggest that FD therapy may be better suited for non-saccular aneurysms within the M1 trunk or M2 segments rather than traditional bifurcation saccular lesions.

In-stent thrombosis and FD implantation failure may lead to severe complications, particularly in the MCA with compromised collateral circulation. Perioperative monitoring of individual variability in platelet response is critical to ensure successful FD deployment in small-caliber vessels. Our protocol incorporates thromboelastography and CYP2C19 genetic polymorphism testing to optimize antiplatelet therapy for patients with poor metabolizer phenotypes or inadequate platelet inhibition. For non-saccular aneurysms with fusiform or elongated morphology, FD placement may require anchoring the proximal end in the ICA and the distal end in the M2 segment of the MCA. The marked discrepancy in luminal diameters between these regions complicates accurate FD length estimation post-deployment. Selecting an FD device based solely on proximal parent vessel diameter risks incomplete stent expansion and malapposition within the narrower MCA. Pre-procedural stent simulation software is therefore invaluable for device selection and procedural planning (7). In this study, preoperative virtual stent simulations were utilized for 27 cases (62.8%) with complex anatomy, enabling precise device selection and reducing intraoperative challenges. No in-stent thrombosis events occurred in this study cohort, highlighting the critical role of tailored antiplatelet regimens and preemptive management strategies.

### Limitations and future directions

This study has several limitations, including its retrospective design and small sample size, which may constrain the generalizability of the findings. Second, the absence of long-term data precludes definitive conclusions regarding FD durability and branch vessel patency. Future research should prioritize prospective studies employing standardized imaging protocols—such as high-resolution vessel wall magnetic resonance imaging—to systematically assess endothelialization processes and hemodynamic alterations. Third, compared with previous studies, our cohort had a relatively shorter follow-up duration, and the long-term clinical outcomes remain unclear. Furthermore, proximal MCA non-saccular aneurysms frequently involve the entire vessel circumference, causing the morphology of the FD within the lesion segment to be significantly influenced by the operator's push-pull maneuvers. And the parent artery centerline simulated by the Aneuguide^TM^ software may not coincide with the actual stent centerline. Last but not least, comparative investigations evaluating FD against alternative therapies (e.g., bypass-assisted clipping) are also warranted to establish optimal treatment algorithms and refine clinical decision-making.

## Conclusion

Proximal MCA non-saccular aneurysms of the represent rare cerebrovascular pathologies for which conventional therapeutic approaches entail substantial technical challenges. Preliminary case series suggest that FD treatment demonstrates favorable safety and efficacy profiles, with optimal device selection and precise deployment serving as critical determinants of procedural success. However, the long-term implications of FD placement on covered branch vessels necessitate extended follow-up to assess potential hemodynamic consequences.

## Data Availability

The raw data supporting the conclusions of this article will be made available by the authors, without undue reservation.
